# PM2.5 induced cardiac hypertrophy via CREB/GSK3b/SOS1 pathway and metabolomics alterations

**DOI:** 10.18632/oncotarget.25479

**Published:** 2018-07-20

**Authors:** Kuan-Lun Li, Yen-Chang Lin

**Affiliations:** ^1^ Graduate Institute of Biotechnology, Chinese Culture University, Taipei, Taiwan

**Keywords:** particulate matter, blood pressure, action potentials, polysaccharides, electrocardiography

## Abstract

The particle matter with diameter less 2.5μm (PM2.5) easier to adsorb toxic substance, and interfere with pulmonary gas exchange. In this study, cardioprotective effects of low molecular weight (LMW) fucoidan in cardiac hypertrophy subjects induced by PM2.5 exposure was conducted by measuring QT interval, Blood pressure, cardiac structure, metabolites and proteins expression in different organs. After PM2.5 exposure, increase in blood pressure, abnormal cardiac function (Prolongation of Action Potential Duration and QT Interval), and structral remodeling (cardiac hypertrophy and fibrosis) were recorded. Fucoidan supplement in consecutive 28 days can reduce the damage to myocardial injury caused by PM2.5. Clearance effect of fucoidan in serum, heart, kidney, lung and liver was found due to organic and inorganic compounds reduced SOS1, CREB, GSK3b, and GRB2 protein level were changed under PM2.5 exposure. Whereas, only CREB level was reduced after fucoidan treatment. Metabolic alteration was also determined that PM2.5 severely damage cardiac tissue and compromise its function. After treatment with fucoidan, the cardiac function was significantly recovered. Our finding demonstrated that LMW could enhance the cardiac status of mice with PM2.5 exposures by rescued QT interval prolongation, action potential and cardiac hypertrophy, and cardiac fibrosis decline.

## INTRODUCTION

Particle matter (PM)is any particle suspended in the air. According to the size of the particle, it is classified into PM10, PM2.5, and PM1.0 with particle size smaller than 10 μm, 2.5 μm, and 1 μm, respectively [1–3]. According to an updated report of American Heart Association (AHA), deaths related to cardiac malfunction and PM2.5 exposure are significantly associated [4]. In previous control-exposure studies, black carbon, titanium dioxide or vanadium pentoxide was used as sole exposure source of PM2.5, however, they cannot reflect the complex mixture of real PM2.5 which is varied according to location and time [5]. The current strategy is to use so-called concentrated ambient particles (CAPs) to get rid of above problems [5].

Fucoidan has been identified firstly by Kylin [6]. It can be extracted from various types of brown algae [7, 8]. Depending on the extraction method and algae species, fucoidan biological and chemical characteristics would differ from one another [9]. Fucoidan has been extensively studies recently in anti-cancer property and others areas [10–13]. The proportion of sulfate and molecular weight of fucoidan are highly associated with its biological function [14, 15]. The small molecular weight of fucoidan (MW < 30kDa) has high biological function and vice versa [16–18]. The cardioprotective effects of fucoidan was demonstrated previously [19, 20]. This study aims to investigate on the underlying mechanism of LMF in ameliorating cardiac function afterconsecutive 28 days exposed to PM2.5 in rodent subjects.

## RESULTS

### Inorganic compounds in CAPs PM2.5

We detected the concentration of inorganic compounds (heavy metal) in PM2.5, such as Cd, As, Pb, Cu, and Hg by using iCAP -Q ICP-MS (Thermo Fisher Scientific, Waltham, MA, USA).

**Figure 1:**
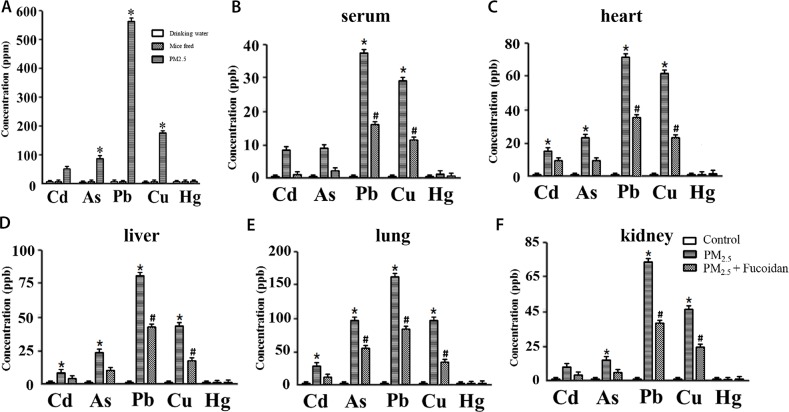
Concentration of 5 heavy metals in mice drinking water, feed, and PM2.5 **(A)**. Concentration of 5 heavy metals in serum **(B)**, heart **(C)**, liver **(D)**, lung **(E)**, kidney **(F)** of 3 groups (Control, PM2.5, and PM2.5 + Fucoidan). PM2.5 had significantly higher concentration of Pb, Cu, and As relative to drinking water and feed. These results show that PM2.5 is the source of heavy metal compounds which may affect functions of heart and other organs (^*^ for comparison with drinking water and feed, the values displayed as mean ± SD, P <0.05).

Our results indicated that Pb had the highest concentration (560±1.59 ppm) in PM2.5. Cu as well as As were also had higher concentrations (180±1.36, and 94.5±2.04 ppm) (Figure 1A).

### Inorganic compounds in mice serum and organs

Heavy metals (Cd, As, Pb, Cu, and Hg) in mice serum and organs were detected by iCAP -Q ICP-MS (Thermo Fisher Scientific, Waltham, MA, USA). The concentration of heavy metal in clean is Pb < 100 ng/m3, As = 1.5-53 ng/m3, Cu < 200 ng/m3. Our results indicated that concentration of 5 inorganic compounds decreased after fucoidan treartment.

Inorganic compound concentration in serum, heart, kidney, lung, and liver were all high in Pb, and Cu. After fucoidan supplement, the concentration of Pb, and Cu decreased siginificantly (Figure 1B–1F).

### H&E staining and masson's trichrome staining

**Figure 2:**
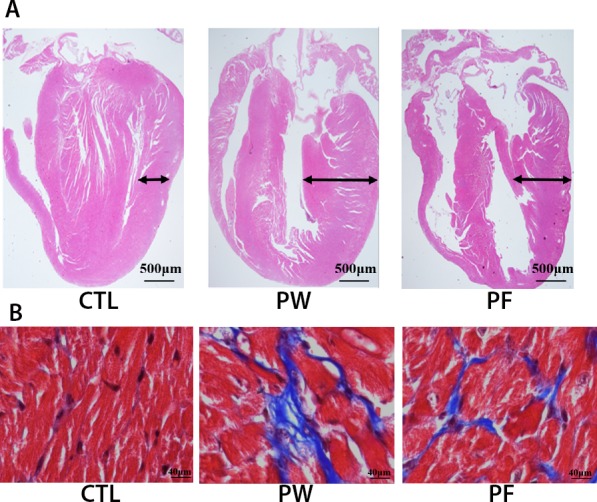
H&E staining for cardiac morphology **(A)** and Masson's trichrome staining for cardiac fibrosis **(B)**.

The heart hypertrophy and fibrosis induced by PM2.5 were observed in PW group but not in control group. After 28 days treatment with fucoidan both heart morphology and fibrosis of subjects were significantly ameliorated (Figure 2A, 2B).

In summary, histological examination findings might considerably clarify the malfunction of cardiac system such as QT interval and action potential alteration via structural changes in mice exposed to PM2.5 and these changes could dramatically reverse to almost normal condition when treated with LMF.

### Effect of LMF on systolic and diastolic pressure in PM2.5-exposed mice

**Figure 3:**
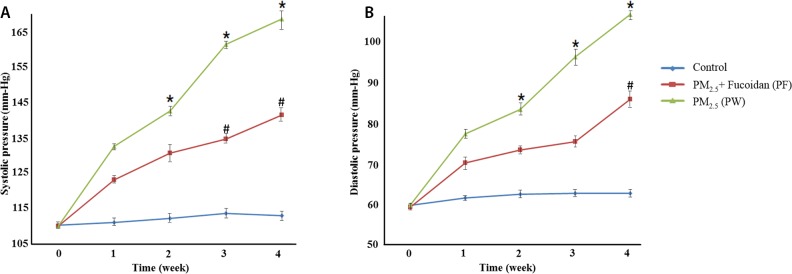
Systolic pressure measurements for 4 weeks **(A)**. Compared with PM2.5 exposed mice and control group, mice with supplemented fucoidan had a significant decrease in systolic and blood pressure. Diastolic pressure measurements for 4 weeks **(B)**. Compared with PM2.5 exposed mice and control group, mice with supplemented fucoidan had a significant decrease in diastolic blood pressure as well. ^*^ for comparison with Control; # for comparison with PM2.5, the values were mean ± SD, P <0.05.

The results of systolic and diastolic pressure in PM2.5-exposed mice, treated with LMF shown significant reduction relative to control group. In the beginning of this experiment, there were no differences among all 3 groups. Besides, systolic and diastolic pressure all reduced after LMF treatment (systolic pressure at week 4, Ctrl:111.4±0.89, PW:167±1.82, and PF:139.9±2.28; diastolic pressure at week 4, Ctrl:62.8±1.3, PW:105±1.64, and PF:85.1±1.14) (Figure 3).

### Effect of LMFon QT interval and Action Potential in PM2.5-exposedmice

**Figure 4:**
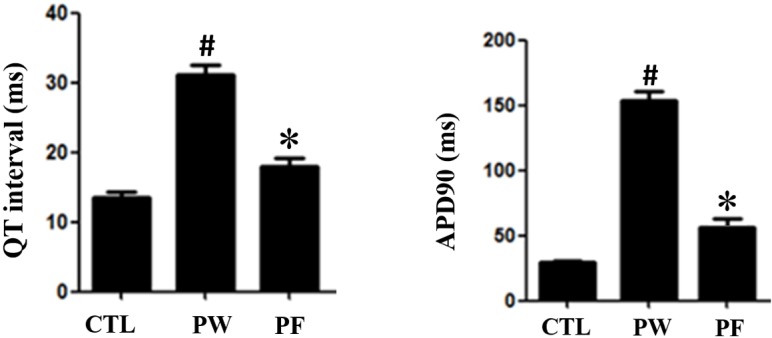
ECG of each group QT interaval in PM2.5 exposure mice had prolongation. However, after fucoidan treatment, ECG prologation significiantly decreased (^*^for comparison with Control; # for comparison with PM2.5, the values were mean ± SD, P <0.05). Action potential of mice ventricular myocyte. The plateau phase had the same trend to ECG (^*^for comparison with Control; # for comparison with PM2.5, the values were mean ± SD, P <0.05).

Prolonged QT interval was determined in PM2.5-exposed group (PW) (0.062688±0.0049)relative to control group (0.039532±0.0036). QT interval reduced when treated with LMF (PF300:0.043126±0.0065). Consistently to the results of QT interval prolonged in PM2.5-exposed model, the action potential duration in myocytes of mice in PM2.5 group shown significant increment relative to control group. Action potentialdecreased in PM2.5-exposed mice treated with LMF (Figure 4A, 4B). The alteration of action potential found in ventricular myocytes possibly due to ionic mechanisms.

### Metabolic analysis

**Figure 5:**
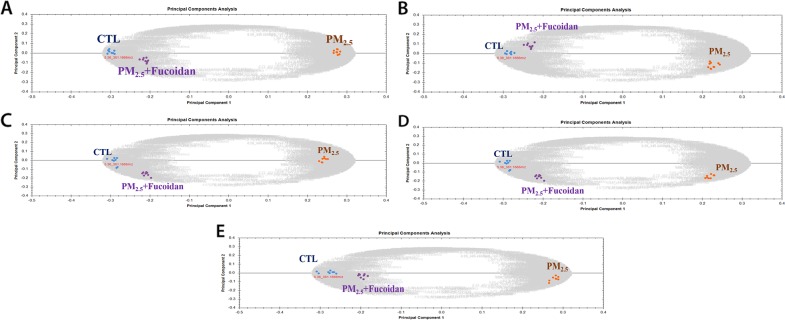
Principle component analysis of metabolites from three groups **(A)** Metabolites from serum sample, **(B)** Metabolites from heart sample, **(C)** Metabolites from kidney sample, **(D)** Metabolites from lung sample, **(E)** Metabolites from liver sample.

Metabolic changes in PM2.5-exposed mice serum and organs (heart, liver, lung, and kidney) extracts were analyzed by using UPLC-Q-TOF MS system and its software Progenesis QI (Waters). First, we used Principal Component Analysis (PCA) to investigate grouping of metabolites among different groups (Figure 5). In order to identify potential compounds as biomarkers, we choose top 5 metabolites via Fragmentation Score. The results showed that 1) top 5 metabolites in serum are Benzo(a) pyrene, Niacinamide, Phenanthrene, Acenaphthene, and Citrate; 2) top 5 metabolites in heart are Hippurate, Arginine, Ornithine, Neryl rhamnosyl-glucoside, N-(1-Deoxy-1-fructosyl) alanine; 3) top 5 metabolites in lung are Lysophosphatidylcholine (24:0), Sphinganine (17:0), Sulfatide, Glucosylceramide (24:1), Sulfatide (12:0) ; 4) top 5 metabolites in liver are N (6) -(1,2-dicarboxyethyl) AMP, Glycyl-Tryptophan, Adenosine 2′-phosphate, TG(18:0/18:0/18:0), 4′-MeSO2-polychlorinated biphenyls 87; 5) top 5 metabolites in kidney are Hypoxanthine, Hippurate, 3-Octanedione, Phenylglyoxylic acid, Allantoin. (Table 1).

**Table 1:** Top 5 metabolites in mice serum, heart, lung, liver, and kidney after PM2.5-exposed

Metabolites in serum		Metabolites in heart		Metabolites in lung	
Compound Name	Fold Change	Compound Name	Fold Change	Compound Name	Fold Change
Benzo(a) pyrene	2.8	Hippurate	4.2	Lysophosphatidylcholine (24:0)	8
Niacinamide	2.2	Arginine	2.6	Sphinganine (17:0)	1.8
Phenanthrene	3.1	Ornithine	2.1	Sulfatide	10.3
Acenaphthene	4	Nerylrhamnosyl-glucoside	3.3	Glucosylceramide (24:1)	1.9
Citrate	3.7	N-(1-Deoxy-1-fructosyl) alanine	28.6	Sulfatide (12:0)	3.4
Metabolites in liver			Metabolites in kidney		
Compound Name		Fold Change	Compound Name		Fold Change
N (6) -(1,2-dicarboxyethyl) AMP		2.8	Hypoxanthine		2.5
Glycyl-Tryptophan		2.5	Hippurate		18
Adenosine 2′-phosphate		6.1	2,3-Octanedione		2
TG(18:0/18:0/18:0)		3.1	Phenylglyoxylic acid		3.7
4′-MeSO2-polychlorinated biphenyls 87		12	Allantoin		2.2

All metabolites were sorting via fold change.

### SOS1, CREB and GSK3b protein level were elevated under PM2.5 exposure

**Figure 6:**
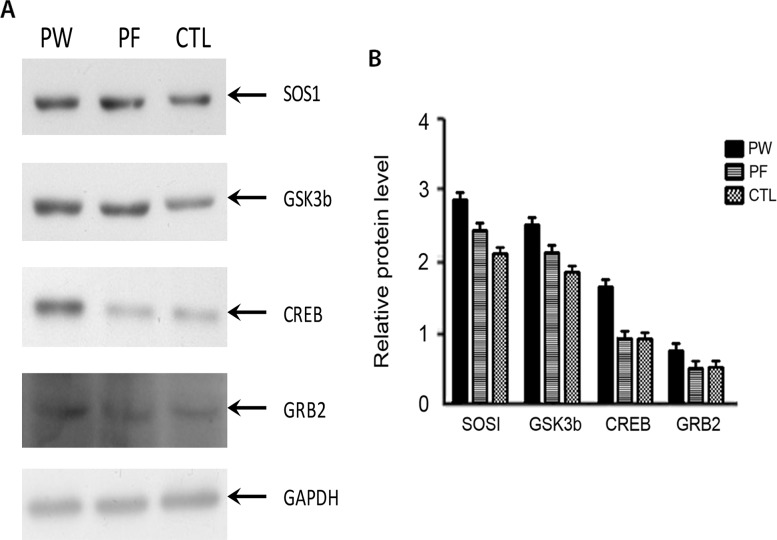
Western blot results of 4 proteins namely SOS1, GSK3b, CREB and GRB2 from three groups **(A)**. Quantification bar chart of SOS1, GSK3b, CREB and GRB2 protein level **(B)**. CTL: control, PW: PM2.5 exposed subjects treated with normal water, PF: PM2.5 exposed subjected treated with fucoidan) treated with normal water, PF: PM2.5 exposed subjected treated with fucoidan.

In order to figure out potential mechanism of how fucoidan ameliorated cardiac function of PM2.5 exposed mice, proteins expression level of SOS1, CREB, GRB2, GSK3b were investigated. Western blot data shown SOS1, CREB and GSK3b expression levels were increased while GRB2 were not changed under PM2.5 exposure (Figure 6). CREB level was reduced under fucoidan treatment while GSK3b, SOS1, and GRB2 were not changed.

### Metacore analysis for potential pathways involve in cardiac hypertrophy

**Figure 7:**
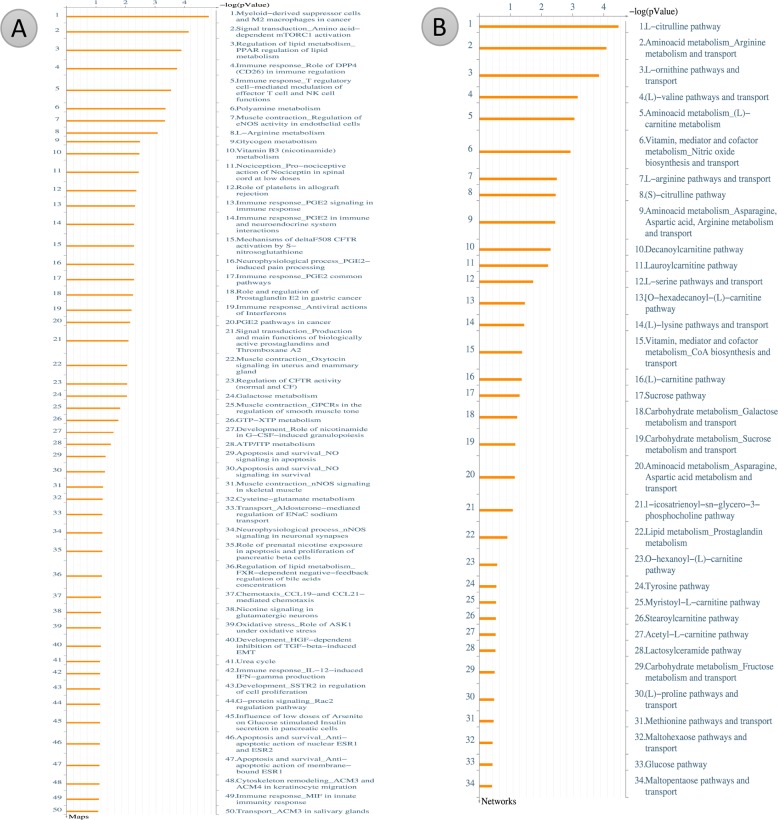
Metacore analysis for potential pathways involve in cardiac hypertrophy **(A)** Pathway Maps analysis. **(B)** Metabolic Networks analysis.

**Figure 8:**
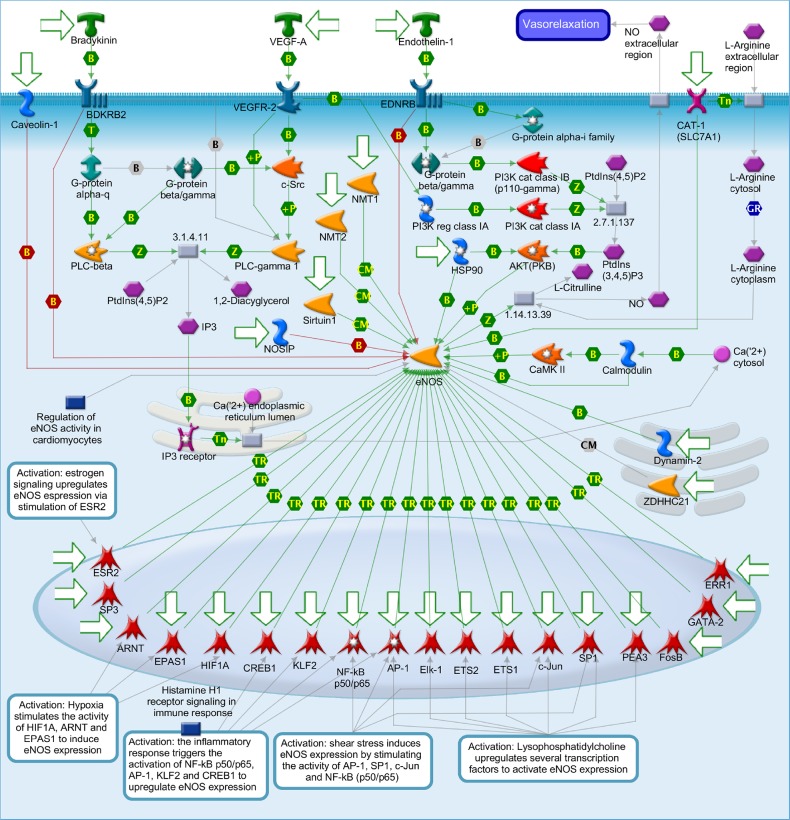
Muscle contraction_Regulation of eNOS activity in endothelial cells

**Figure 9:**
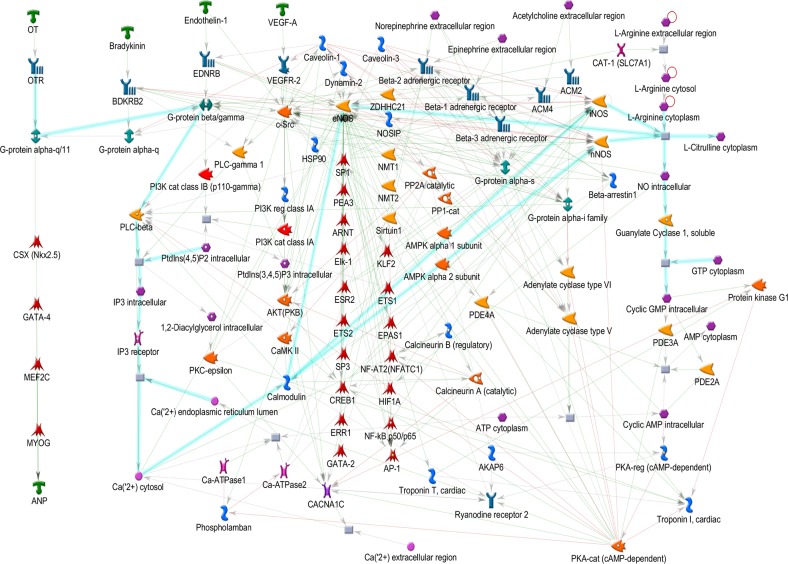
Muscle contraction_Nitric oxide signaling in the cardiovascular system

After metabolites identification, these small molecules were mapped into their related signal pathways by Metacore network database (GeneGo, MI, USA). The results form Metacore shown that “Muscle contraction_Regulation of eNOS activity in endothelial cells” pathway and “Muscle contraction_Nitric oxide signaling in the cardiovascular system” network were likely involved in PM2.5 exposure (Figures 7–9) [21]. Significant difference level was set at p-value< 0.05.

Nitric oxide (NO) is known to involve in catalyzing for the conversion of L-arginine to L-citrulline. NO belongs to family of nitric oxide synthases (NOSs) [21]. There are three manin type of NOSs namely endothelial NOS (eNOS), neuronal NOS (nNOS) and inducible NOS (iNOS) which have different functions. Each of them only expressed in specific types of tissue. For instance, eNOS expressed dominantly in endothelial cells and cardiac myocytes. The nNOS mainly expressed in brain and skeletal muscle as well as cardiomyocytes. The iNOS expressed in a wider range of tissue namely endothelial cells, cardiomyocytes, and inflammatory cells. Under some certain circumstance such pathological condition, eNOS and n NOS can be consider as regulated expression [21]. These two type of NOSs are Ca(2+)/Calmodulin-dependent enzymes that inducible by stimulus- or agonist. In endothelial cells, eNOS generates NO which in turn takes part in angiogenesis and normal blood pressure balancing. Relaxation event of vessel wall is a result of No induced by Bradykinin, Endothelin-1 and VEGF-A in endothelial cell. The activity of eNOS in under control of various cell levels transcription, post-transcriptional and post-translational modifications, such as acylation and phosphorylation, protein-protein interactions, and subcellular localization. Both oxygen consumption and timing for relaxation stage in myocardium are affected by the releases of NO from the coronary endothelium in paracrine mode. In addition, The modulation of basal inotropy and cardiac muscle relaxation are affected by NO produced in the cardiomyocyte in autocrine mode. There are two main group of signals namely neurotransmitters and hormones control the force and frequency of myocardial contraction. Neurotransmitters such as (L)-Noradrenaline and (L)-Adrenalineare released by the sympathetic nerves in the heart and adrenal glands released into the circulation, respectively. These signals rise myocardial contractility of the heart. To reduce the contractility of heart muscle, parasympathetic nerves releases Acetylcholine which binds to the receptor named muscarinic cholinergic. NO is the regulator of these two pathways. NO is generated endogenously within cardiac muscle cells. NO also can affect to various ion channels in the heart. For instance, NO level can modulateL-type Ca(II) channel activity. In some cases, cGMP-dependent Protein kinase G and cGMP-regulated phosphodiesterases can alsomediate NO function [21].

The bioinformatics results suggest that PM2.5 and fucoidan are supposed to participate in the cardiac hypertrophy pathway via eNOS and CREB related signaling (Figures 7–9). Until now, no holistic approach has been used to demonstrate the potential signal pathways involving in PM2.5 exposure and fucoidan effects. Therefore, our work is the first paper to reveal the potential signal pathway may involve in PM2.5 exposure and use fucoidan as bioactive compound to enhance and/or recover the overall cardiac function of exposed subjects. The present investigation provides potential target genes or protein in PM2.5 exposure and fucoidan effects for prospective researches.

## DISCUSSION

In the present study, cardioprotective effects of fucoidan has been demonstrate with various examinations. Fucoidan could likely reduce the cardiac hypertrophy, cardiac fibrosis, long QT interval in PM2.5 exposed mice. Cardiac abnormality by PM2.5 exposure might due to metabolic disorder in numerous metabolites as well as alteration in gene expression such as SOS1, CREB and GSK3b.

Long QT interval was previously proved associated atherosclerosis [22, 23]. QT interval has been documented as a reliable predictor of ventricular arrhythmias [24]. Moreover, Long QT interval, cardiac hypertrophy and cardiac sudden death were highly associated [25]. In the present findings, we found that both ventricular hypertrophy and QT prolongation presented inPM2.5 exposed subjects, which could suggest that PM2.5-exposed mice are more likely facing with cardiac sudden death. However, cardiac function of mice with PM2.5 exposure could be recovered to some extend such as QT recovery and cardiac tissue enhancement, decrease in fibrosis percentage by treatment with fucoidan.

Fucoidan is reported with anti-oxidative and anti-inflammatory properties [26–30]. Recently, accumulation studies focused on the low molecular weight fucoidan due to its higher biological activities [26, 31–34]. Fucoidan also were used to myocardial infarction in rats model, and it shown cardioprotective effect by recovering the damage region induced by isoproterenol [20]. Fucoidan can protect the damage caused by myocardial ischemia-reperfusion [35].

Glycogen synthase kinase-3 (GSK-3) plays vital role in cardiac hypertrophy. In previous study, 2,5-dimethylcelecoxib (DM-celecoxib) can activate GSK-3α and β by inhibiting Akt, to prevent left ventricular hypertrophy and fibrosis [36]. CREB is a well know protein that involves in many crucial pathways related to cardiac fibrosis [37, 38], cardiac hypertrophy [39, 40]. Both GSK3β and CREB protein expression level were up regulated in PW group relative to control group. After fucoidan treatment, CREB level was reduced while GSK3b was not significantly changed. This result suggests that CREB protein may be a target of fucoidan molecule.

Previous studies claimed that PM2.5 caused damage to zebrafish embryo such as skin aging and oxidative stress [41]. Besides, PM2.5 exposure is associated with metabolic diseases such as insulin resistance [42]. In addition, PM2.5 affects to lipoproteins and causes atherosclerosis and embryonic toxicity as well [42, 43]. PM2.5 exposure also results in systemic vascular failure by NADPH oxidase and TLR4 pathways, which cause systemic inflammation. In addition, PM2.5 is also suggested to disturb the reduced-oxidized balance and lead to vasoconstrictive responses [43]. According to our results, we could establish PM2.5-induced high blood pressure mice animal, which can be used as one animal model to identify whether some natural compounds have reversed effects on cardiac dysfunction.

PM2.5 exposure and cardiac abnormality and complications have been reported previously [44]. A 10 μg/m^3^ increase of PM2.5 level within 24 h will increase about 1.0% of the relative risk (RR) of daily cardiovascular mortality [45]. According to our study, cardiac structural remodeling and dysfunction caused by PM2.5 were recorded. Besides, metabolomics was used as the main approach to identify component of PM2.5 which will highly impact to cardiac function, and pathway they patriciate. First, we found benzo[a]pyrene (B[a]P), a well-known carcinogenic polycyclic aromatic hydrocarbon (pAH), has the highest level in PM2.5 exposure mice serum, may cause DNA damage in lung adenocarcinoma CL-3 cells. Based on related research report, 10 mM B[a]P-treated CL-3 cells have G2/M arrestwhich was independent with p53 pathway. In addition to B[a]P, we found out other 2 compounds, acenaphthene and phenanthrene, which belong to PAH group as well.

B(a) P was reported to cause defect of cardiovascular development by aryl hydrocarbon receptor or up regulated of rbp4 and may results in cardiac hypertrophy by activating TLR4/MyD88 signal pathway [46–48].

Our finding demonstrated that LMW could enhance the cardiac status of mice with PM2.5 exposures by rescued QT interval prolongation, action potential and cardiac hypertrophy, and cardiac fibrosis decline. The *in vitro* and *in vivo* toxicology of PM2.5 and its mode of action were not thoroughly investigated. The elements of PM2.5 mixture varies from one to another area. Therefore, it is important to investigate the individual and combinatory impact of PM2.5 compounds.

## MATERIALS AND METHODS

### Animal model

Total 24 C57BL/6J mice, 8 weeks old, were purchased form (BioLasco company, Taiwan. Mice were housed in animal house in standard conditions with light/dark period of 12h each. Food and water were always available for animals. Three groups of animals, each of 8, according to experimental conditions were obtained. They were control group (Ctrl) with no PM2.5 exposure or fucoidan (Hi-Q Oligo-Fucoidans^®^) treatment, PM2.5 exposed group without treatment (PW), and PM2.5 exposed group with 300mg/kg fucoidan administration daily in 28 days (PF). The study procedure with animal was approved by IACUC, Chinese Culture University, Taiwan, ROC.

### CAPs PM 2.5 preparation and exposure to animal

The BGI PQ200-FRM Sampler machine was used for PM2.5 collection at Taipei Main Station 3 hours per day from November to December, 2016. Mice were exposed to PM2.5 solution (100μg/m^3^), which is the collected PM2.5 powder in ddH_2_O, in a chamber. The flow rate of PM2.5 solution is 3.5-4.0 L/min, and the solution is atomized with the mixture of air. All the mice exposed to PM2.5 consecutive 28 days (6h/day) and every 5 days took a break of 2 days. ECG and blood pressure were measured every week. After 28 days mice were all sacrificed.

### CAPs PM 2.5 composition analysis

We used UPLC-Xevo G2-S Q-Tof (Waters, USA) system and iCAP -Q ICP-MS (Thermo Fisher Scientific, Waltham, MA, USA)to analyze components of PM2.5 mixture.

### Blood pressure measurement

BP2000 Visitech Systems was used for measure blood pressure from mice tail by an emitter assembly, which contains a red LED to illuminate the tail. Before started, all the mice should put into the Specimen platform, which holds the animals, and has the sensor(s) and occlusion cuff(s). Blood pressure of each mouse was detected for 10 times every weekend. All the data recorded by the BP-2000 Analysis software.

### ECG recording

ECG measurement was done to record the QT interval of experimental mice. ECG system consist of 3 leads vector connected to the signal recording and application system (PowerLab/4SP analog-to-digital converter, AD Instruments, Colorado Springs, CO). A mixture of 2% isoflurane and compressed oxygen was used for anesthesia with flow rate at 2L/min [49, 50]. Data of each group were derived from 8 mice.

### Action potential recording

The method was previously described in detail [51]. In brief, action potential from ventricular isolated cardiomyocyte from mice was recorded in Tyrode and pipette solutions. The patch clamp setup was done with bright-field and fluorescent light sources, and CCD camera.

### Histology (H&E staining)

Hematoxylin and eosin staining method was described previously by Phan et al. [52]. Briefly, the whole heart was removed and washed twice in PBS to remove the blood as much as possible. It was then fixed in 4% paraformaldehyde (PFA) at 25^°C^ overnight. PFA section then exchanged with 70% Ethanol, fixed in paraffin and cut at 2.5 mm. Paraffin sections were rehydrated and stained with hematoxylin and eosin.

### Masson trichrome staining

Masson trichrome staining was done as our previous publication [52]. Masson trichrome staining was done as our previous publication [28]. Briefly, alcohol (100% alcohol, 95% alcohol 70% alcohol) was used to de-paraffinize and rehydrate cardiac tissue. To improve staining quality, Bouin's solution was applied for 15 minutes at 56^°^C and rinse under tap water for 5-10. Next, immerged in Weigert's iron hematoxylin solution 10 minutes then wash with PBS buffer 5 minutes. Afterward, stained with Biebrich scarlet-acid fuchsin solution for 5 minutes, then washed in distilled water. Then sections underwent series of differentiation, dehydration in 75% and 90% alcoho and rinsing in tap water. Finally, sections were cleared in xylene and mounted.

### Western blot assay

This assay was done as previously described document [52]. Briefly, the cardiac tissue was isolated and removed all fat and connective tissue. Tissue homogenization was done with lysis buffer (Thermo Fisher Scientific Inc.) on ice. Centrifugation was used to remove large tissue and nuclear fragments at 7000 g, 4°C for 10 minutes then collected supernatant. NANOVUE PLUS™ SPECTROPHOTOMETER (Harvard Bioscience, Inc.) was used for protein concentration measurement. Total protein (40 μg) was loaded to 10% SDS gel and run for 1h at 100 mA. Afterward, a polyvinylidine fluoride (PVDF) membrane was used to transfer protein bands on ice in 1h at 100 mA. Blocking of membrane was done with 10% non-fat dry milk (Bio-rad) and then incubated overnight with primary antibodies diluted in 5% milk. Primary antibody as followed: polyclonal antibodies against Phospho-GRB2 antibody [Y237] (ab32037) (1:1000, ABCAM), Phospho-SOS1 (ab64595) (1:1000, ABCAM), phosphor-GSK3 beta [Y216] (ab75745) (1:1000, ABCAM), phosphor-CREB [E113] (ab32096) (1:1000, ABCAM) and GAPDH (1:1000, ABCAM). Afterward, secondary antibodies, horseradish peroxidase (HRP) (1:10000), was used to incubate with the membranes at room temperature. The membrane was finally read by C-DiGit® Blot Scanner (LI-COR Biosciences).

### Metabolomic analysis

#### Sample preparation

Upon sacrifice, serum was collected. The whole heart was acquired rapidly and washed with PBS twice for blood removal and freeze in liquid nitrogen. Liver, lung, and kidney tissue were also collected and immerged in liquid nitrogen. All organs were then stored at −80^°^C for later experiment.

To prepare serum for mass spectrometry analysis, protein precipitationmust be done with cold acetonitrile (ACN). For sample preparation of tissue from organs, 100 μl RIPA buffer with 1μl phosphatase inhibitor was added to 10 mg tissue of each organ and ultra-sonicated for 1 min. 400 μlof ACN was added into 100 μl of homogenate and centrifugedat 12,000 g for 15 min, at 4°C. The supernatant was obtained andfiltered with 0.45 μm filter for mass spetrometry analysis.

#### LC-MS/MS analysis

For metabolomics analysis, UPLC-Q-TOF MS system (Waters) was employed. Samples were injected to Acquity UPLC BEH C18 column (2.1×50 mm, 1.7 μm; Waters) by 0.1% formic acid (FA) in water. Elution process was done with gradient of 0.1% FA in CAN at flow rate of 0.2 mL/min for 10 min for each sample. Electrospray ionization (ESI) positive mode was chosen for Q-TOF. Metabolite mass per charge ratio was set from 100 to 1,500. Finally, MassLynx software (Waters) was employed for mass accuracy processing and composition of the precursor and fragment ions analysis.

#### LC-MS/MS data processing

Raw LC/MS data extracted from MassLynx software (Waters) was processed Progenesis QI software for Metabolite identification. Theoretical fragmentation analysis was used for metabolite identification. Fragmented data were matched by default databases of Progenesis QI (Human Metabolome Database (http://www.hmdb.ca) and ChemSpider database (www.chemspider.com). Principle Component Analsysis figures was generated by Progenesis QI.

### Metacore analysis for potential pathways involve in cardiac hypertrophy

After metabolite identification, these small molecules were mapping to their related signal pathways. Accordingly, we used Metacore network database (GeneGo, MI, USA) pathways of these compounds. The gene-metabolites interaction was also included in the analysis. MetaCore generated biological networks from the list of identified metabolites. Comparing the metabolites of PM2.5 exposed samples and PM2.5 unexposed samples, we uploaded the significantly metabolites into Metacore software. The results form Metacore shown that “Muscle contraction_Regulation of eNOS activity in endothelial cells” pathway and “Muscle contraction_Nitric oxide signaling in the cardiovascular system” networkwere likely involved in PM2.5 exposure (Figures 7–9). Significant difference was set at p-value< 0.05.

### Statistical analysis

One-way analysis of variance (ANOVA) for statistical different between group was done by SPSS version 20. Data were displayed in mean ± standard deviation. *P-value* < 0.05 means significant different.

## Abbreviations

RR: Relative Risk
GSK-3: Glycogen Synthase Kinase-3
DM-Celecoxib: 2,5-Dimethylcelecoxib
ESI: Electrospray Ionization
ACN: Acetonitrile
PVDF: Polyvinylidine Fluoride
PFA: Paraformaldehyde
Ctrl: Control Group
PW: PM2.5 Exposed Group Without Treatment
PF: PM2.5 Exposed Group With 300 Mg/Kg Fucoidan Administration Daily In 28 Days
Caps: Concentrated Ambient Particles
PM: Particle Matter
LMW: Low Molecular Weight
